# Prices Current

**Published:** 1865-10

**Authors:** 


### PRICES CURRENT.

It is curious to compare the prices current as issued by the various manufacturers of what, in contradistinction to chemicals, are called pharmaceuticals. Take for instance the class of Fluid Extracts, one list before us quotes Fluid Extract of Sarsaparilla (Comp.) at $2.85 per lb.; another quotes the same at $1.25. One quotes Fluid Extract of Buchu at $3.25, another the same at $1.25, and so on, the prices varying from twice to three times as much on one list as on another. Now a fluid extract, according to every acknowledged authority, is made, with a very few well-known exceptions, of such strength that for every troy ounce of the drug there must be just a fluid ounce of the Fluid Extract. This is the strength prescribed by the U. S. Pharmacopœia, and that which of course forms the basis of their employment in the treatment of disease, and especially in making the other preparations of the same drug. Where a troy ounce of a drug is used in making a pint of its syrup, a fluid ounce of its fluid extract added to fifteen fluid ounces of simple syrup, would be expected to furnish a pint of syrup of corresponding strength. So simple and easy is this method of preparing the weaker liquid preparations used in medicine, that it is now very much resorted to by Pharmaceutists and Physicians, and is recognized in several instances in the Pharmacopœia. What, then, is the reason that Fluid Extracts differ so greatly in price from different makers? Is it because some possess facilities for making them so much cheaper than others, or must we look for other causes? This, we think, can be answered conclusively thus: Throwing out of view the exact processes laid down in the Pharmacopœia, to which some manufacturers conscientiously adhere, there are only two considerations which can materially affect the economy of extraction of drugs—these are the completeness of the means of pressure and of the recovery of the excess of alcohol employed. At the present enormous cost of alcohol it is essential that not a drop shall be wasted to secure an economical result; hence most skilful percolation must be resorted to, connected with pressure for securing every drop of the percolate, and then well adjusted apparatus for distillation must be used in concentrating the liquid. These requirements are met, perhaps, by all the leading manufacturers, and yet this great disparity in prices. The true reason is, undoubtedly, that some few manufacturers make these preparations of full strength, while the majority wholly ignore the standards of the Pharmacopœia, and sell preparations of just such strength as suits themselves. We will not ask whether this is justifiable on the ground of professional ethics, but we put it to the reader whether it is dealing justly towards that very large class of purchasers who are led by names, with very little knowledge as to the real merits of a preparation. If a fluid extract may represent one-fourth its weight of a drug, or may be equal to its own weight, according to the whim of the maker, what value can it have in any rational scheme of medicine? As to the interest of the purchaser, it evidently lies in this, as in all other cases, in getting the best. If a man will ignore the standards of good practice in his manipulations, what confidence can one have in his fairness in business transactions? On the other hand, inflexible honesty in manufacturing indicates a similar principle in dealing. We should not think it necessary to go over these obvious points, but for the purpose of calling attention to an invariable rule which may be applied to these price currents. When a Fluid Extract is offered at a given price, let the buyer count up the cost of the drug, remembering that a pint will take sixteen troy ounces (equal to a commercial pound and near two ounces), with the menstruum, almost always alcoholic, and which by every process is liable to some waste, and the sum of these items will be the minimum cost of the preparations. To get at the fair selling price, a margin must be added for fuel and labor, and a profit of not less than twenty per cent. to the manufacturer. If the price quoted falls much below this, depend upon it there is a deficiency somewhere, and the apparent cheapness does not imply economy to the purchaser.

### CARBUNCLE.

Our readers may remember an appeal addressed to the public by a French parish priest, in order to obtain funds for the publication of a pamphlet, in which an infallible cure for carbuncle was to be revealed. This plan seems to have been abandoned for the simple one of publishing the remedy in one of the medical journals; for we find in the *Union Medicale* an article by Dr. Topinard, in which he describes the Dardelle secret as follows:—"Prepare a round piece of linen of a sufficient size to cover the whole diseased part, and spread thereon a slight film of storax ointment, and then a layer of corrosive sublimate (be-chloride of mercury) of the thickness of a two-franc piece. The plaster thus prepared is laid with the greatest care upon the part affected, and kept in its place with strips of sticking-plaster. After twenty-four hours this plaster may be removed, and it will then be infallibly found that the carbuncle or pustule has been destroyed. The place must now be dressed three times a day with storax ointment spread upon linen; and at every dressing the part must be fomented with a mixture of the oil of linseed, cammomile, and hypericum. In the course of eight or ten days the eschar falls off, and the sore is treated like a common one."

This remedy, discovered by a blacksmith of the name of Dardelle, has never been known to fail. Dr. Miso, from whom the prescription has been obtained, has used it these ten years with invariable success; and Dr. Topinard concludes with reason that sublimate exercises a specific action in such cases. It is the more desirable that such a remedy should be widely circulated, since this very morning we find a new case of a man at Annouville-Vilmesnil, near Fecamp, who, having been stung by a venomous fly, neglected applying proper remedies, and the consequence is that his finger has had to be amputated to prevent gangrene. He is now doing well.—*Galignani.*

---

INFANTILE SYPHILIS.—The use of mercurials is quite unnecessary. Chlorate of potash given in two-grain doses thrice daily is all the medicinal treatment required. The nose usually requires well syringing with warm water. (Mr. R. W. Dunn, p. 224.)

### AN ENGLISH CURE FOR DRUNKENNESS.

There is a prescription in use in England for the cure of drunkenness, by which thousands are said to have been assisted in recovering themselves. The receipt came into notoriety through the efforts of John Vine Hall, commander of the Great Eastern steamship. He had fallen into such habitual drunkenness, that his most earnest efforts to reclaim himself proved unavailing. At length he sought the advice of an eminent physician, who gave him a prescription which he followed faithfully for several months, and at the end of that time he had lost all desire for liquor, although he had been for many years led captive by a most debasing appetite.

The receipt, which he afterwards published, and by which so many other drunkards have been assisted to reform, is as follows:—Sulphate of iron, five grains; magnesia, ten grains; peppermint water, eleven drams; spirit of nutmeg, one dram; twice a day. This preparation acts as a tonic and stimulant, and so partially supplies the place of the accustomed liquor, and prevents that absolute physical and moral prostration that follows a sudden breaking off from the use of stimulating drinks.—*Eclectic Med. and Surg. Journal.*

### SUCCESSFUL SURGICAL OPERATION.

The French papers tell of the success of a curious surgical operation, heretofore considered impossible. A young girl, turning her head too quickly, dislocated some of the up per cervical vertebræ. A paralysis of the lower limbs and the trunk ensued; the diaphragm alone retained its sensation. Apparently the patient had but a few hours to live. M. Mais sonneuve resolved to attempt to bring the vertrebræ into the former position. With the assistance of a fellow-surgeon, he seized the patient by the top of the head and the chin, and gave a gentle rotary motion to the vertebræ, which resulted in bringing them into their former position. The paralysis ceased almost immediately, and a week after the patient was able to walk as though nothing had happened to her.

